# Differences in Subtidal Macrobenthic Community Structures and Influencing Factors Between Jindo and Jeju Islands in South Korea

**DOI:** 10.1002/ece3.70990

**Published:** 2025-02-24

**Authors:** Jian Liang, Chae‐Woo Ma, Kwang‐Bae Kim

**Affiliations:** ^1^ Department of Biology, College of Natural Sciences Soonchunhyang University Asan Republic of Korea; ^2^ Research Group of Tidal Flats Gyeonggi‐Do Maritime and Fisheries Resources Research Institute Ansan Republic of Korea

**Keywords:** anthropogenic activities, distance‐based redundancy analysis, heavy metal, macrobenthos, Southern Sea of Korea

## Abstract

Although islands in South Korea have been significantly impacted by human activities, marine ecological studies focusing on island coasts have been limited. Due to their distinct characteristics, macrobenthos is extensively utilized to assess the impact of anthropogenic influences on the marine environment. In August 2010, August 2011, and September 2012, samples of macrobenthic communities, bottom water, and sediment were collected from the subtidal zones around southern Jindo and northern Jeju islands in South Korea. Macrobenthos was identified to the species level using a stereomicroscope. Bottom seawater quality was evaluated, with a focus on dissolved heavy metal concentrations (As, Cr, Cd, Cu, Pb, and Zn). Additionally, we measured the organic matter content and mean grain size of the sediment. There were marked differences in macrobenthic community structures between the two islands, including the number of species, species abundance, species richness index, and Pielou's evenness index (*p* < 0.05). Cluster analysis, non‐metric analysis, permutational multivariate analysis of variance (PERMANOVA), and permutational analysis of multivariate dispersion (PERMDISP) revealed variations in macrobenthic communities between the two islands and over the years 2010, 2011, and 2012. According to the Biota‐Environment Matching (BIO‐ENV), distance‐based redundancy analysis (dbRDA), and distance‐based linear model analyses (DistLM), the principal environmental variables influencing the distribution of macrobenthic communities are Cd and As. These variations likely result from different levels of human activity on each island. Moreover, interannual variations in macrobenthic communities, especially in 2012, were predominantly influenced by Pb and Cr, likely due to alterations in the influence of the Changjiang (Yangtze River) diluted water.

## Graphical abstract

Human activities have significantly affected South Korean island coasts, with macrobenthic communities serving as key indicators of environmental impact. Studies in 2010, 2011, and 2012 around Jindo and Jeju Islands revealed distinct differences in macrobenthic structures and species diversity, linked to varying levels of heavy metals (e.g., Cd, As, Pb, Cr) influenced by local human activity and interannual changes in the Changjiang diluted water.

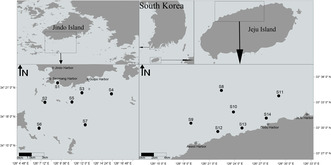

## 1 Introduction

Islands play a critical role in marine ecosystems and offer various socio‐economic benefits to humans (Remoundou et al. 2009; Balzan et al. 2018). They provide essential habitats for wildlife and support industries such as tourism, aquaculture, and other economic activities (Huang et al. 2008). However, the growing global population has increasingly strained island ecosystems in recent decades, with human activities causing significant ecological impacts (Halpern et al. 2019; Rick et al. 2013). These impacts include the loss of native species and the invasion by non‐native species (Geraldi et al. 2020), degradation of terrestrial ecosystems due to urbanization (Chi et al. 2020), and the decline in food diversity for terrestrial invertebrates, driven by the development of island tourism (Steibl et al. 2022). While much research has focused on the effects of human activities on island terrestrial ecosystems (Chi et al. 2020), less attention has been given to the impacts on island marine ecosystems (Fitzpatrick and Giovas 2021).

Macrobenthos, due to their relatively sedentary nature and species‐specific responses to pollution, are widely used as indicators to assess the impact of human activities on marine ecosystems (Hu et al. 2019). Numerous studies have confirmed that macrobenthos serve as effective bioindicators for evaluating heavy metal pollution in sediments (Izegaegbe et al. 2023; Rabaoui et al. 2015). However, research on the effects of dissolved heavy metals in seawater on macrobenthos remains limited. Heavy metal pollution in seawater has long been a concern due to its impact on marine ecosystems and the risks it poses to human health through bioaccumulation in the food chain (Zhang et al. 2020; Li et al. 2022). Studies have shown that the accumulation of heavy metals in the tissues of crustaceans, mollusks, and fish correlates with the concentration of these metals in seawater (Gao et al. 2021; Liu et al. 2021). Although recent studies suggest that consuming fish and invertebrates caught along South Korea's coast does not pose a significant health risk from heavy metal intake (Mok et al. 2014; Hwang et al. 2017), the impact of heavy metals in South Korean seawater on marine ecosystems remains understudied.

South Korea is an industrial powerhouse, concentrating heavy industries like shipyards and steel mills in coastal areas. Numerous studies have indicated that industrial activities in the West Sea and South Sea of South Korea have resulted in heavy metal contamination of marine sediments, causing irreversible damage to marine ecosystems (Ra et al. 2013; Song et al. 2014; Kim et al. 2020; Choi et al. 2012). Jeju and Jindo islands, the first and third largest islands in South Korea, cover areas of 1845 km^2^ and 334 km^2^, respectively, and are located in the South Sea. Jeju Island is a globally renowned tourist destination, whereas Jindo Island serves as a significant aquaculture hub in the region. To our knowledge, the impact of heavy metals on the macrobenthic communities of these islands has not yet been studied. The objectives of this study are to (1) evaluate the concentrations of heavy metals in the subtidal waters surrounding Jindo and Jeju Islands, (2) investigate the structure of macrobenthic communities in the subtidal zones of the two islands, and (3) assess the impact of heavy metals on these communities.

## 2 Materials and Methods

### 2.1 Study Area

**FIGURE 1:**
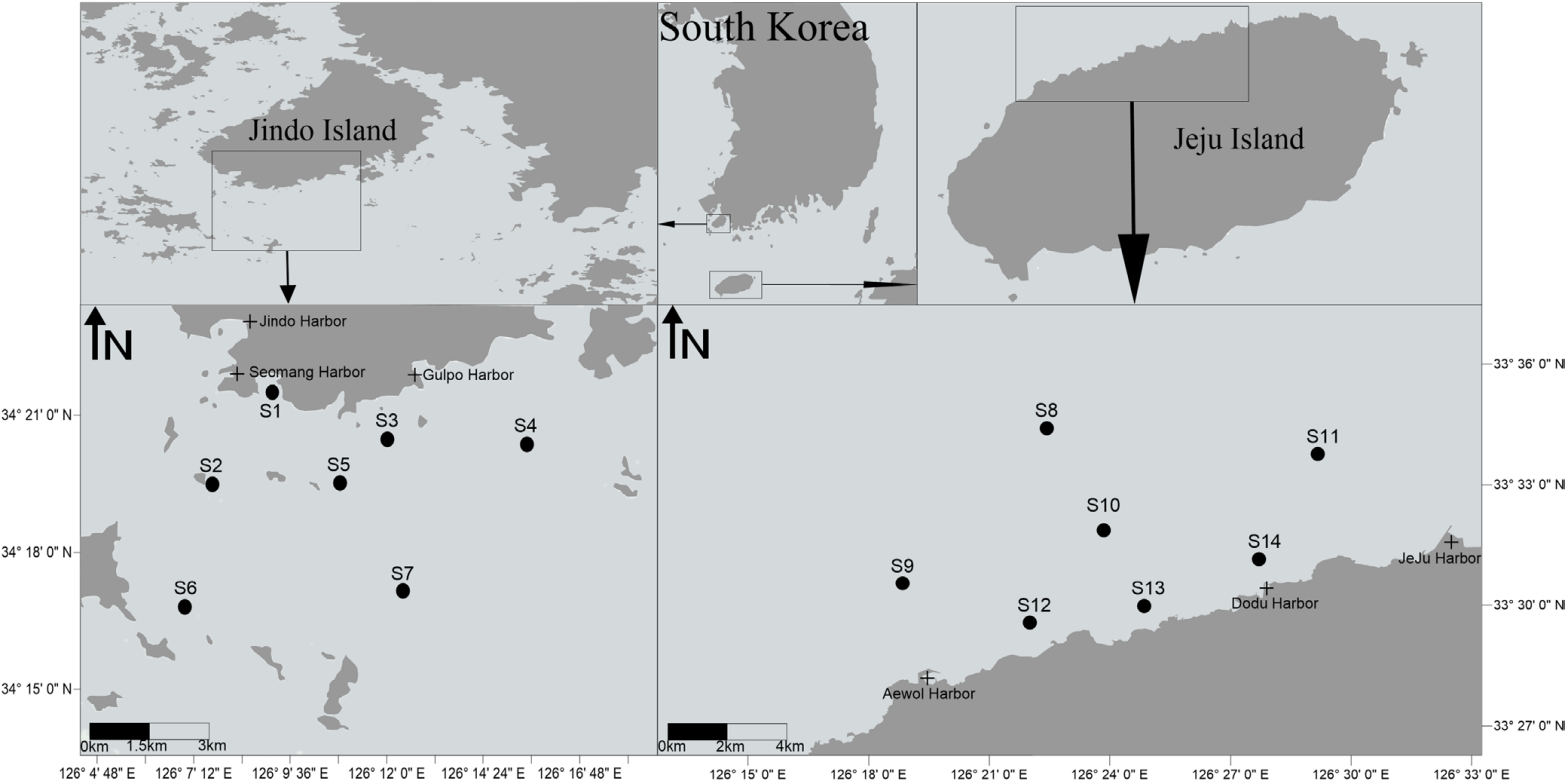
Study areas and sampling sites in the subtidal zones off Jindo and Jeju island, South Korea (2010–2012).

The study areas are located in the subtidal zones off southern Jindo and northern Jeju islands. Jindo Island's southern region features three harbors: Jindo Harbor, Seomang Harbor, and Gulpo Harbor. Similarly, the northern region of Jeju Island consists of three harbors: Aewol Harbor, Dodu Harbor, and Jeju Harbor (Figure 1). Numerous fish farms are located along the southern coast of Jindo Island, while the northern coast off Jeju Island has no such facilities (http://www.khoa.go.kr/oceanmap/main.do#). The seawater temperature off Jindo Island ranges from 5.7°C to 24.2°C, with salinity levels between 30.7 and 36 PSU (Lim and Lim 2016). Conversely, the seawater temperature off Jeju Island varies from 14°C to 20°C, with salinity levels ranging from 33.6 to 34.4 PSU (Kim et al. 1998).

### 2.2 Sampling Collection and Processing

Sample collection occurred in August 2010, August 2011, and September 2012, establishing fourteen sampling sites off Jindo (S1‐S7) and Jeju island (S8‐S14) for each collection period (Figure 1). The coordinates of the sampling sites are shown in Table S1. At each site, samples were collected using a 0.1 m^2^ Van Veen grab, with sediment samples collected once and macrobenthic samples collected twice. Additionally, bottom water samples were collected using a Rosette sampler, and parameters such as temperature, salinity, pH, and dissolved oxygen (DO) were measured using a YSI 6920 multiparameter sonde (YSI Inc., Yellow Springs, OH, USA). Each site's macrobenthic samples were sieved through a 0.5 mm mesh and preserved in 10% neutral formalin solution, while sediment samples were stored in a deep freezer for subsequent analysis. A 2 L sample of bottom water was stored directly in the freezer, and an additional 2 L sample was filtered using glass‐fiber filter papers. Both the filtered water and the filter paper were kept in the deep freezer for future analysis.

In the laboratory, macrobenthic samples were identified to the species level and quantified using a dissecting microscope (SMZ‐168, Motic Ltd., China). Dissolved heavy metals (As, Cr, Cd, Cu, Pb, and Zn) in the bottom water were measured by placing 400 mL of seawater into a 500 mL Teflon separatory funnel, adjusting the pH to approximately 4.5 using an ammonium acetate buffer. A 1 mL mixture of organic chelating agents (APDC/DDDC) was then added and agitated for 10 s. Subsequently, 10 mL of chloroform was incorporated and stirred at 300 rpm for 5 min. After resting for 10 min, the organic solvent layer was separated and collected in a Teflon beaker. The solvent was evaporated by heating at 80°C until dry, and the residue was decomposed with concentrated nitric acid. Finally, 1–2 mL of diluted nitric acid was added, and the heavy metal content was analyzed using inductively coupled plasma mass spectrometry (Agilent 7700, Agilent Inc., USA). Suspended solids were quantified by filtering and drying 200 mL of bottom seawater with glass fiber filter paper. Total Nitrogen (TN) and Total Phosphorus (TP) levels in bottom seawater were determined using colorimetric and spectrophotometric methods with an automated analyzer (QuAAtro SFA, Seal Analytical Ltd., UK). A 50 g sediment sample was dried at 80°C for 48 h and subsequently heated in a muffle furnace at 550°C for 4 h to measure ignition loss (IL). The concentrations of acid volatile sulfide (AVS) and chemical oxygen demand (COD) in the sediment were measured using COD titration and AVS detection tube methods. The wet sieving method was applied to analyze the mean grain size of the sediment. All analyses of seawater and sediment samples complied with the Notification of Marine Environmental Process Test Standards (National Institute of Fisheries Science 2010), and each test was performed in triplicate to ensure accuracy.

### 2.3 Data Analyses

Principal component analysis (PCA) was utilized to evaluate the environmental characteristics of the subtidal zones off Jindo and Jeju Islands. The dominance index identified the dominant species off these islands, with a species deemed dominant if its dominance value exceeded 0.02 (Table S2) (Xu and Chen 1989). The dominance index is widely used to evaluate dominant species in macrobenthic communities within the Yellow Sea (Li et al. 2023). The cutoff value of 0.02 is based on long‐term monitoring studies conducted by regional researchers, which identified this threshold as indicative of ecologically significant dominance patterns. Cluster analysis, non‐metric MDS analysis, and four ecological indices (Table S2) were employed to assess variations in the macrobenthic communities between the two islands (Clarke et al. 2014). The Analysis of Similarities (ANOSIM) determined whether significant differences existed between the groups identified in the cluster analysis.

To determine if there were differences in the number of species, species abundance, and four ecological indices between the two islands, the Shapiro–Wilk test was initially used to check for normality. If the data followed a normal distribution, the independent samples *t*‐test was applied; otherwise, the Mann–Whitney *U*‐test was used. Permutational multivariate analysis of variance (PERMANOVA) was employed to evaluate the effects of island, year, and month on subtidal benthic communities off the islands. However, it could not determine whether the observed differences were driven by location (factor effects) or dispersion (variance). To address this, permutational analysis of multivariate dispersion (PERMDISP) was performed as a post hoc test (Mulik et al. 2020). Redundancy Analysis (RDA) examined the relationship between dominant species and environmental variables, while Spearman rank correlation analysis evaluated the relationships between the number of species, species abundance, and four diversity indices with environmental variables. A distance‐based linear model (DistLM) was employed to explore the relationship between macrobenthic communities and various environmental factors. Distance‐based redundancy analysis (dbRDA) visualized the fitted model, and stepwise regression identified an optimal subset of environmental factors. Biota–Environment Matching (BIO‐ENV) analysis determined the optimal combination of environmental variables that best explained the influence on macrobenthic community structure.

PCA, cluster analysis, non‐metric multidimensional scaling (MDS) analysis, ANOSIM, PERMANOVA, PERMDISP, DistLM, dbRDA, and BIO‐ENV were conducted using PRIMER 7.0 software (PRIMER‐E, Wellington, New Zealand). RDA was performed using Canoco 5 software (http://www.canoco5.com). Spearman rank correlation analysis and the Shapiro–Wilk test were executed using Origin 2021 software (OriginLab Inc., Northampton, USA). Independent samples t‐tests and Mann–Whitney U tests were carried out using IBM SPSS 29.0 software (SPSS Inc., Chicago, USA). The Spearman rank correlation analysis heatmap was generated using Origin 2021 software. Blue represents negative correlations, while red indicates positive correlations. The deeper the color, the greater the absolute value of the correlation coefficient.

## 3 Results

### 3.1 Environmental Characteristics in Study Areas

Range and mean values of environmental factors in study areas are shown in Tables S2 and S3. Off Jindo Island, the highest coefficient of variation was observed for acid‐volatile sulfide at 0.77, whereas off Jeju Island, it was for As at 1.16 (Tables S3 and S4). The average concentration of heavy metals in seawater off Jindo Island followed the sequence: Pb (0.035 μg/L) < Cd (0.036 μg/L) < Cr (0.078 μg/L) < As (0.094 μg/L) < Cu (0.266 μg/L) < Zn (0.343 μg/L); off Jeju Island, it was: Cd (0.017 μg/L) < Pb (0.018 μg/L) < As (0.026 μg/L) < Cr (0.038 μg/L) < Cu (0.101 μg/L) < Zn (0.255 μg/L).

**FIGURE 2:**
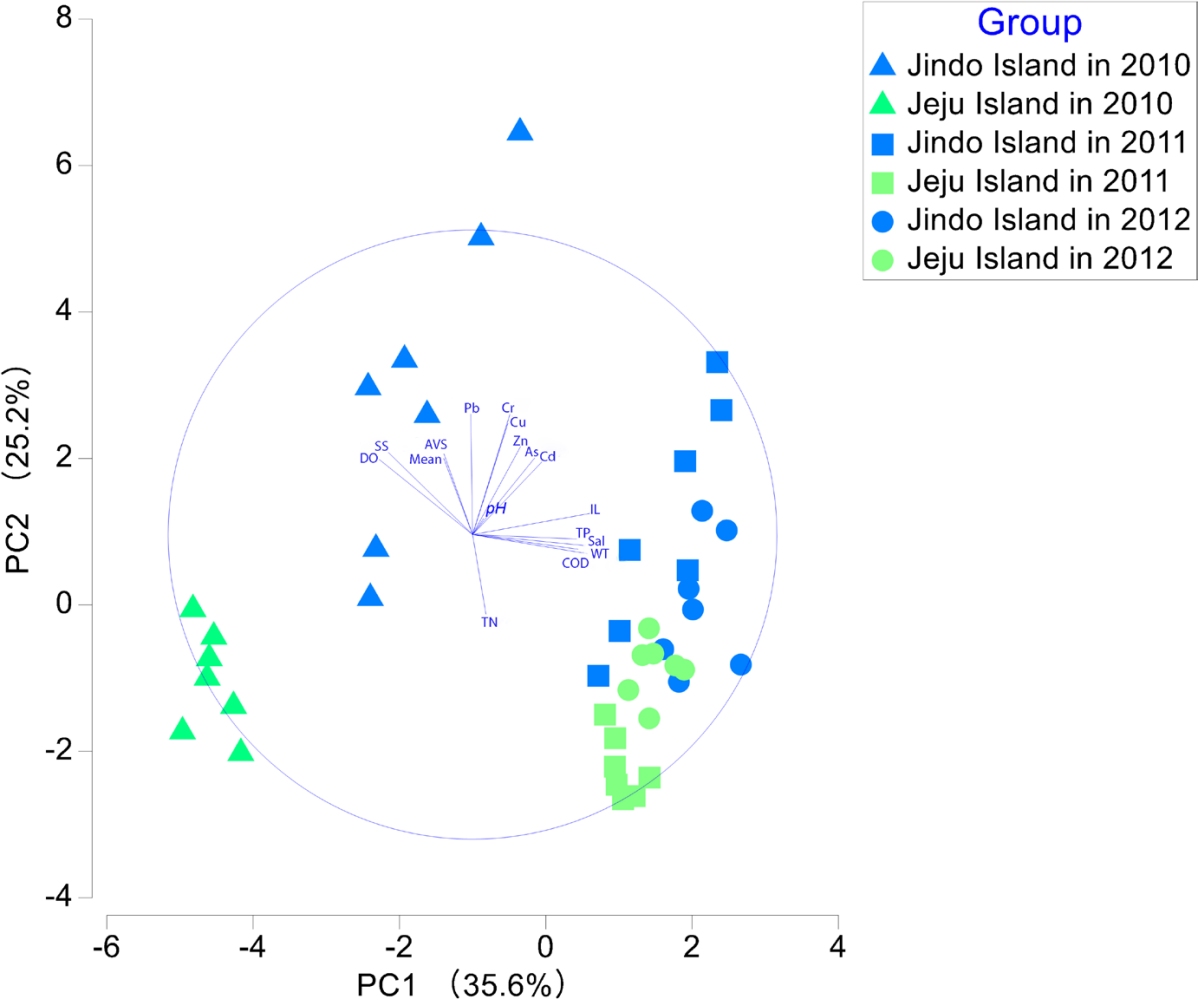
PCA of environmental factors in the subtidal zones off Jindo and Jeju island. AVS, acid‐volatile sulfide; COD, chemical oxygen demand; DO, dissolved oxygen; IL, ignition loss; Mean, mean grain size; N, species abundance; S, species number; Sal, salinity; SS, suspended solids; TN, total nitrogen; TP, total phosphorus; WT, water temperature.

In the PCA, the PC1 and PC2 axes accounted for 35.6% and 25.2% of the variance in environmental factors, respectively (Figure 2). The PC1 axis primarily reflected organic matter characteristics in sediments and seawater salinity, while the PC2 axis mainly represented the characteristics of heavy metals in seawater. Sampling sites off Jeju Island in 2010 were positioned on the left side of the PCA plot, indicating the lowest levels of chemical oxygen demand and ignition loss compared to other years' sites (Table S5). Contrary to Jeju Island, sampling sites off Jindo Island were generally positioned in the upper region of the plot, suggesting higher concentrations of Cr and Pb in the subtidal zone off Jindo island compared to Jeju Island (Table S5).

### 3.2 Macrobenthic Community Structures Off Jindo and Jeju Island

In this study, 98 macrobenthic taxa were identified in the subtidal zone off Jindo Island, consisting of 58 Annelida species, 17 Mollusca species, 12 Arthropoda species, 6 Echinodermata species, and 5 species from other animal groups. Meanwhile, off Jeju Island, 104 macrobenthic taxa were identified, including 61 species of Annelida, 19 species of Mollusca, 14 species of Arthropoda, 4 species of Echinodermata, and 5 species from other animals.

**FIGURE 3:**
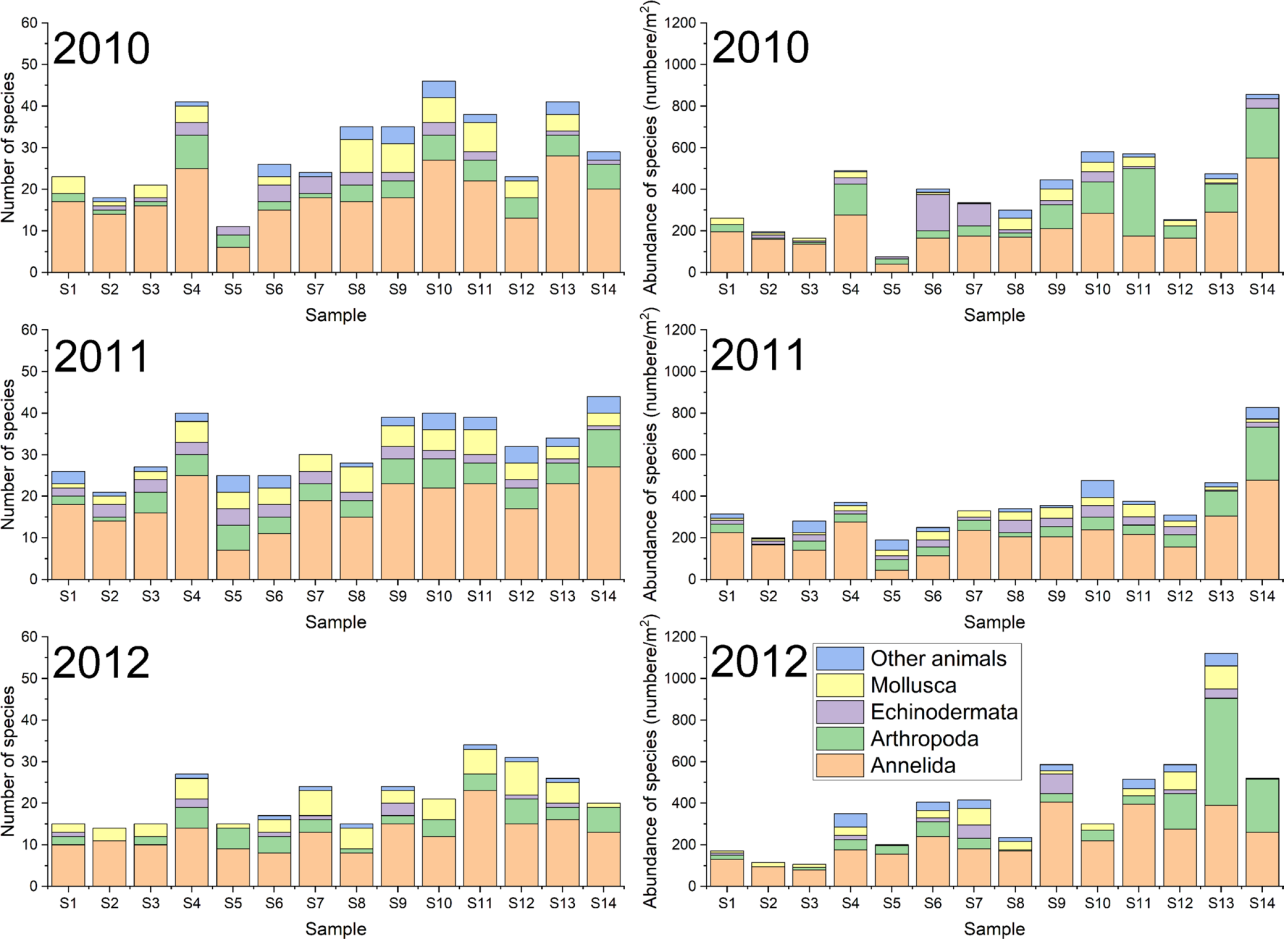
Number and abundance of species at each station in the subtidal zones off Jindo and Jeju island. S1–S7, Sampling sites off Jindo Island; S8 –S14, Sampling sites off Jeju Island.

The highest number of species was recorded at site 4 in 2010, with 115 species, while the lowest was at site 5 in the same year, with 11 species (Figure 3). The highest abundance of species was observed at site 13 in 2012, with 1120 individuals per m^2^, and the lowest at site 5 in 2010, with 75 individuals per m^2^ (Figure 3). Four dominant species were identified in the subtidal zone off Jindo Island, with *Heteromastus filiformis* (polychaeta) showing the highest dominance value of 0.1. Off Jeju Island, three dominant species were identified, with Gammaridea exhibiting the highest dominance value of 0.13 (Table 1).

**TABLE 1:** Dominant species in the subtidal zones off Jindo and Jeju island.

Sample	Taxa	Species	Dominance value
Jindo Island	Annelida	*Heteromastus filiformis*	0.10
	Arthropoda	Gammaridea	0.06
	Annelida	*Ampharete arctica*	0.05
	Annelida	*Prionospio sp*.	0.03
Jeju Island	Arthropoda	Gammaridea	0.13
	Annelida	*Amphicteis gunneri*	0.09
	Annelida	*Ampharete arctica*	0.04

**FIGURE 4:**
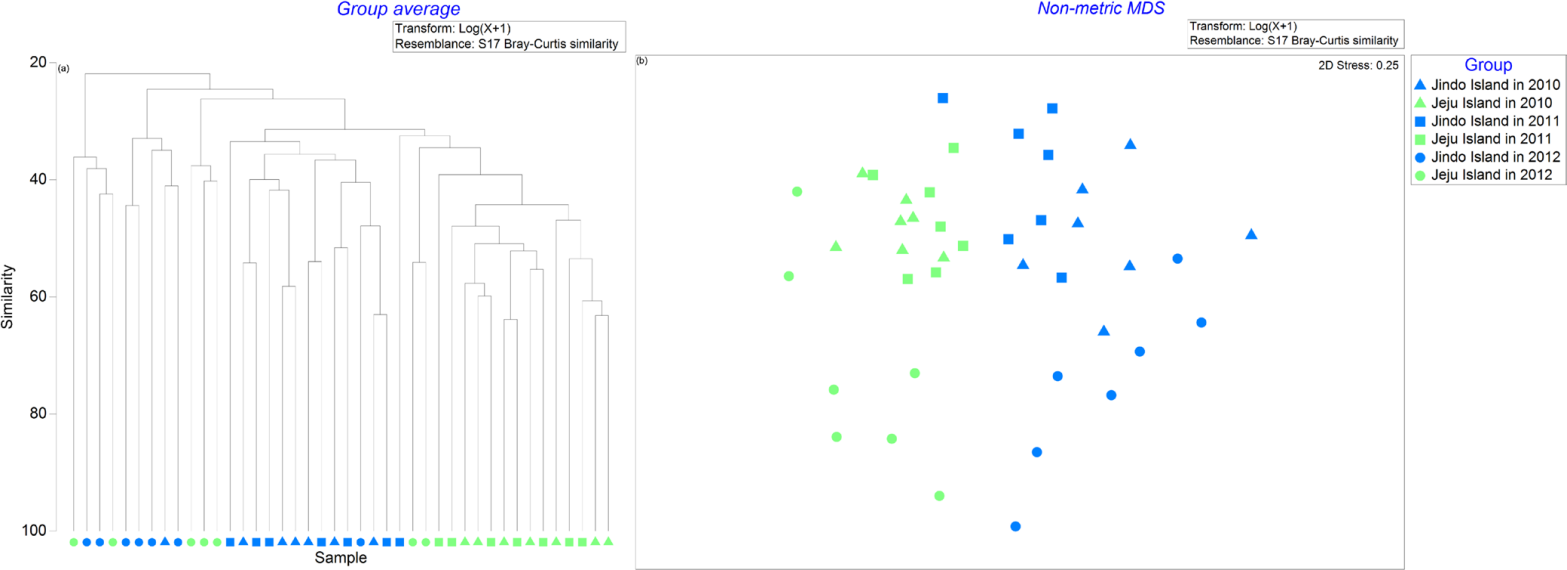
Cluster analysis (a) and Non‐metric MDS analysis (b) based on the macrobenthic community's Bray–Curtis similarity in the subtidal zones off Jindo and Jeju island. The clustering method employed in the cluster analysis was the group average linkage method.

PERMANOVA identified significant differences in macrobenthic community structures among islands (*F* = 3.0315, *p* = 0.001) and across years (*F* = 6.6187, *p* = 0.001). However, differences among months could not be assessed due to variations in group sizes (Table 2). PERMDISP revealed significant differences in dispersion across years (*p* = 0.003) and months (*p* = 0.0009), while no significant differences in dispersion were found among islands (*p* = 0.076) (Table 2). Cluster analysis highlighted distinct differences in benthic community structures in 2012 compared to other years. It also demonstrated high similarity in the benthic communities of Jeju Island in 2011 and 2012 and those off Jindo Island during the same period. Similarly, the MDS plot showed that sample points from Jeju and Jindo Islands in 2011 and 2012 were tightly clustered, whereas the sample sites in 2012 were more dispersed (Figure 4).

**TABLE 2:** Results of PERMANOVA and PERMDISP on macrobenthic communities using island, year, and month as factors.

	Source	Pseudo‐*F*	*p*(perm)		Source	*F*	*p*
PERMANOVA	Island	3.0315	0.001	PERMDISP	Island	3.3178	0.076
	Year	6.6187	0.001		Year	10.092	0.003
	Month	Not test	Not test		Month	13.011	0.0009

### 3.3 Environmental Drivers of Macrobenthic Community Structure Off Jindo and Jeju Island

**FIGURE 5:**
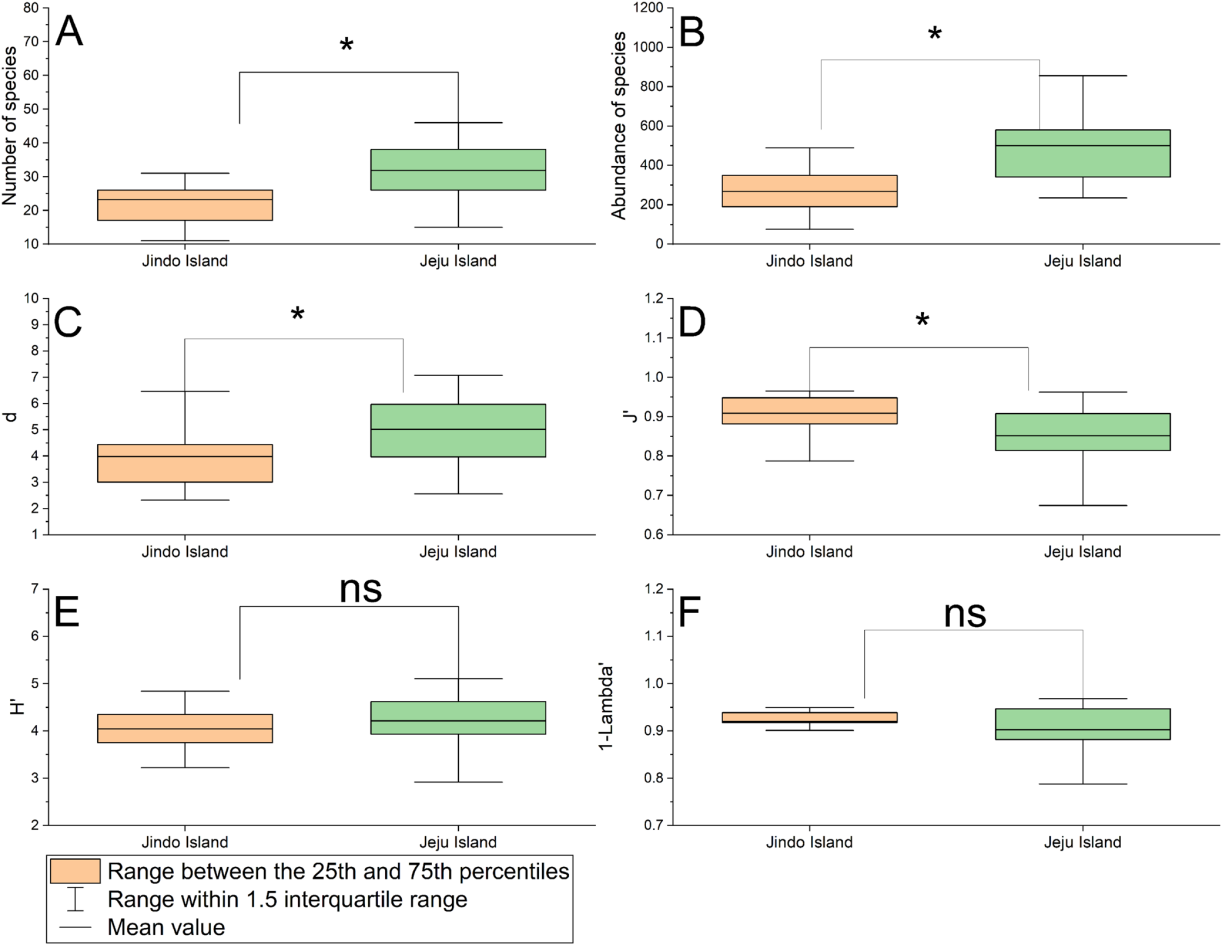
Comparison of the number of species, species abundance, and four ecological indices in the subtidal zones off Jindo and Jeju island. d (C), species richness index; J' (D), Pielou's evenness index; 1‐Lambda' (F), Simpson index; H′ (E), Shannon‐Wiener diversity index; the Mann–Whitney *U*‐test was applied to analyze the number of species (A), J', and 1‐Lambda'; the independent samples t‐test was utilized for examining species abundance (B), d, and H′; *p < 0.05; ns, *p >* 0.05.

The mean values of the number of species, species abundance, and four ecological indices are depicted in Figure 5. The values of the four ecological indices for each site are presented in Figures S1–S4. Except for the mean value of the 1‐Lambda' index, other mean values off Jeju Island were higher than those off Jindo Island. Significant differences were observed in the number of species, species abundance, d index, and J' index between Jindo and Jeju Islands (Figure 5) (*p* < 0.05). Overall, the macrobenthic communities off Jeju Island had higher species diversity, abundance, and species richness compared to those off Jindo Island, highlighting differences between the two islands of macrobenthic communities.

**FIGURE 6:**
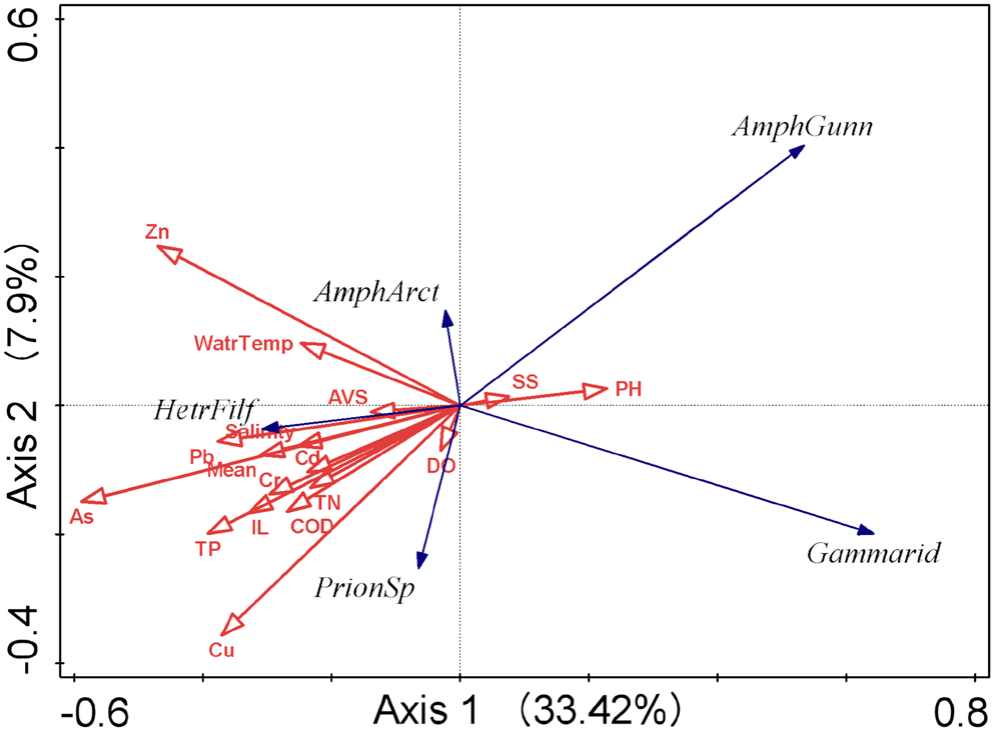
RDA sequence of dominant species and environmental factors in the subtidal zones off Jindo and Jeju island. AVS, acid‐volatile sulfide; COD, chemical oxygen demand; DO, dissolved oxygen; IL, ignition loss; SS, suspended solids; TN, total nitrogen; TP, total phosphorus; Watr Temp, water temperature; Mean, mean grain size; AmphArct, *Ampharete arctica* (Annelida); AmphGunn, *Amphicteis gunneri* (Annelida); Gammarid, Gammaridea (Arthropoda); HetrFilf, *Heteromastus filiformis* (Annelida); PrionSp, *Prionospio* sp. (Annelida).

In the RDA analysis, Axes 1 and 2 together explained 41.32% of the variation in environmental variables (Figure 6). *Amphicteis gunneri* (Annelida) and Gammaridea (Arthropoda) were positively correlated with suspended solids and pH, and negatively correlated with other environmental factors. *Ampharete arctica* (Annelida) showed a positive correlation with Zn and water temperature, but a negative correlation with other environmental variables. Conversely, *Heteromastus filiformis* (Annelida) and *Prionospio* sp. (Annelida) showed negative associations with suspended solids and pH, and positive correlations with other environmental factors (Table 1).

**FIGURE 7:**
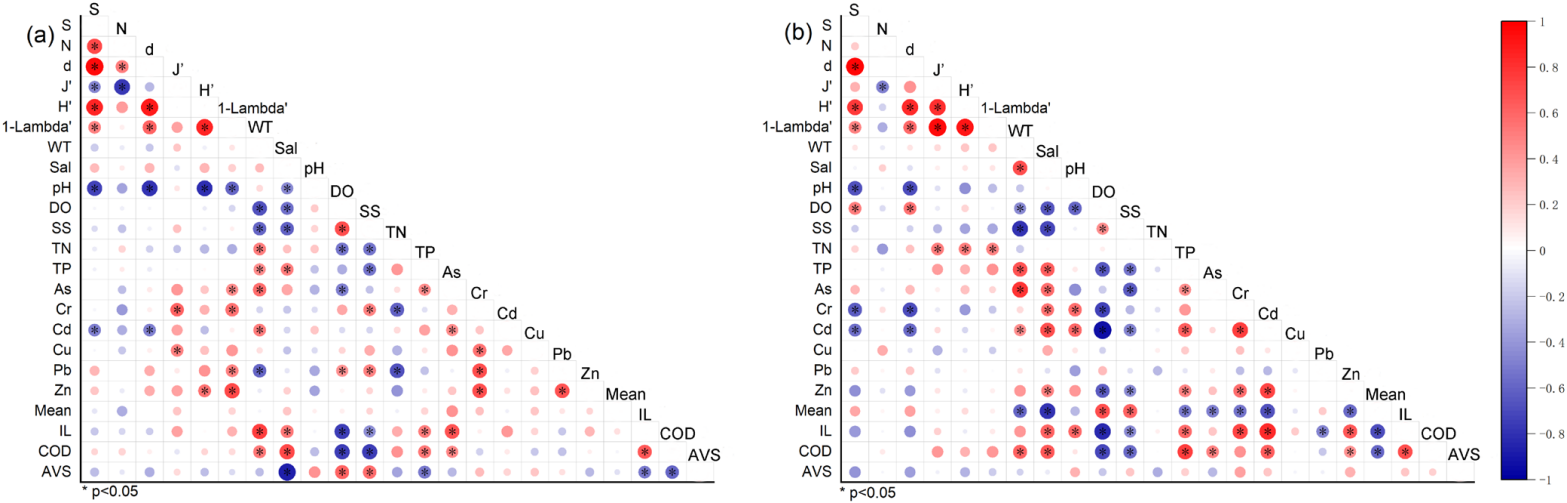
Heatmap of the Spearman rank correlation analysis between species number, species abundance, four diversity indices, and environmental factors off Jindo (a) and Jeju island (b). AVS, acid‐volatile sulfide; COD, chemical oxygen demand; DO, dissolved oxygen; IL, ignition loss; Mean, mean grain size; N, species abundance; S, species number, Sal, salinity; SS, suspended solids; TN, total nitrogen; TP, total phosphorus; WT, water temperature; d, species richness index; J', Pielou's evenness index; 1‐Lambda', Simpson index; H′, Shannon‐Wiener diversity index; *, *p* < 0.05.

In the subtidal zones off Jindo Island, species number (S) was correlated with pH and Cd, while species richness index (d) also exhibited correlations with pH and Cd. J' displayed correlations with Cr and Cu, and H′ showed correlations with Zn. Additionally, 1‐Lambda' was correlated with pH, As, Cr, Pb, and Zn (Figure 7a).

In the subtidal zones off Jeju Island, species number (S) correlated with pH, DO, Cr, and Cd, with species richness index (d) showing correlations with the same factors. J', H′, and 1‐Lambda' correlated with total nitrogen (Figure 7b).

**FIGURE 8:**
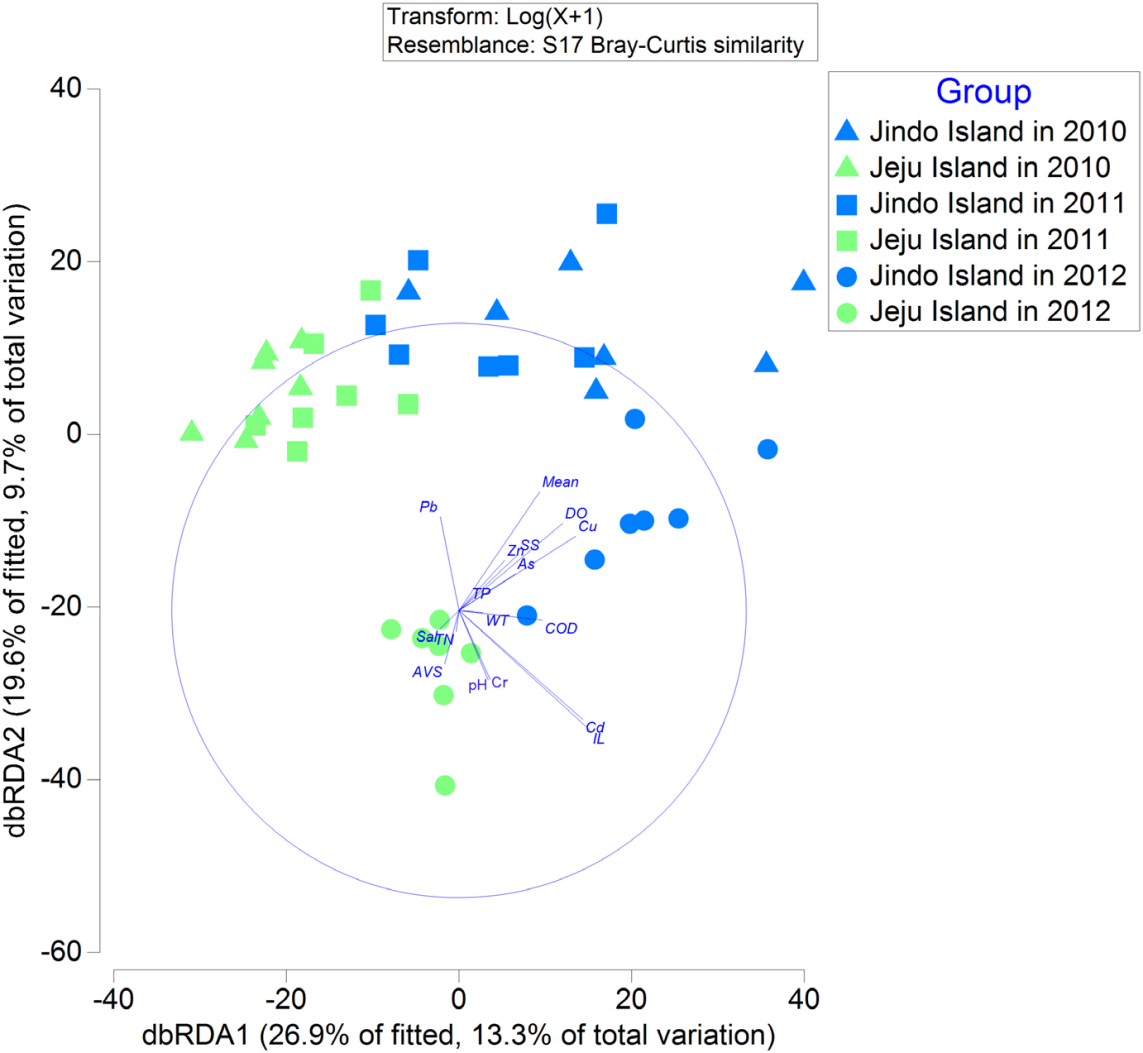
Distance‐based redundancy analysis (dbRDA) ordination plot depicting the relationship between the macrobenthic community and environmental factors in the subtidal zones off Jindo and Jeju island. AVS, acid‐volatile sulfide; COD, chemical oxygen demand; DO, dissolved oxygen; IL, ignition loss; Mean, mean grain size; N, species abundance; S, species number; Sal, salinity; SS, suspended solids; TN, total nitrogen; TP, total phosphorus; WT, water temperature.

In the dbRDA analysis, the dbRDA1 and dbRDA2 axes explained 26.9% and 19.6% of the model's variance, respectively. Combined, these axes accounted for 46.5% of the variation in the data cloud (Figure 8). The dbRDA1 axis was negatively correlated with AVS, salinity, suspended solids, Cr, and Pb but positively correlated with other environmental factors. Similarly, the dbRDA2 axis was negatively correlated with AVS, IL, pH, salinity, As, Cd, and Cr but positively correlated with other environmental factors (Table S6).

The sampling sites off Jeju island are located on the left side of Figure 8, while the sites off Jindo island are on the right side, indicating that COD, DO, Cd, and As are significant environmental factors influencing community differences between the two islands (Table S6). Sampling sites from 2012 are on the downside of Figure 8, while those from 2010 and 2011 are on the upside, showing that COD, DO, Pb, and Cr are key environmental factors influencing community differences across the years (Table S6).

It is significant to note that in the DistLM analysis, Cd (*F* = 5.0, *p* < 0.001), Cu (*F* = 4.0, *p* < 0.001), As (*F* = 3.5, *p* < 0.001), Cr (*F* = 3.3, *p* < 0.001), Pb (*F* = 3.1, *p* < 0.001), IL (*F* = 2.7, *p* < 0.001), pH (*F* = 2.4, *p* < 0.05), Zn (*F* = 2.1, *p* < 0.05), mean grain size (*F* = 2.0, *p* < 0.05), and salinity (*F* = 1.6, *p* < 0.05) were significantly correlated with the distribution of macrobenthic communities.

In BIO‐ENV analyses, in the subtidal zone off Jindo Island, water temperature, pH, Pb, and mean grain size were the environmental variables most strongly correlated with the macrobenthic community structure. Conversely, in Jeju Island, Cr and Cd were strongly correlated with the macrobenthic community composition. As Cd and Pb were the environmental variables most consistently correlated with macrobenthic communities at both locations (Table S7).

## 4 Discussion

### 4.1 Environmental Characteristics in the Subtidal Zones Off Jindo and Jeju Island

In our study, the average concentrations of IL, COD, and mean grain size in the subtidal zones off Jindo and Jeju island were lower than those in Asan Bay, Cheonsu Bay, and Gyeonggi Bay on the western coast of South Korea (Liang et al. 2024b). Additionally, the average concentrations of IL and COD were lower than those in Dangdong Bay (Liang et al. 2024a), located in the southern sea of South Korea, and the average concentration of IL was lower compared to the central part of the East Sea of South Korea (Liang et al. 2024c).

The average concentrations of heavy metals in the seawater around Jindo and Jeju islands were lower than the Korea Marine Seawater Quality Standard and lower than in Gyeonggi Bay, Masan Bay, and the Yellow Sea (Table 3) (Figure S5). However, the average concentrations of six heavy metals in the seawater of southern Jindo were higher than those in northern Jeju Island (Table 3) (Figure S5). This discrepancy is likely due to several factors. First, rivers in South Korea's southwestern coast flow into the sea near Jindo and bring heavy metal pollution from industrial parks, agricultural runoff, and commercial activities (Yang et al. 2020). These pollutants enter the sea, impacting the local marine environment. Second, Jindo Island is in Mokpo Bay, where the slower seawater flow rate hampers pollutant dispersion, resulting in higher concentrations of heavy metals. In contrast, the open waters around Jeju Island experience faster currents, facilitating better dilution. Additionally, compared to Jeju's coast, numerous fish farms along Jindo's coast (http://www.khoa.go.kr/oceanmap/main.do#) might influence metal levels. Some studies indicate that copper‐based antifouling agents used in aquaculture and metals in fish feed can contribute to heavy metal pollution in marine ecosystems (Sapkota et al. 2008; Nikolaou et al. 2014).

**TABLE 3:** Comparison of heavy metal concentrations (μg/L) and average concentrations in seawater between the study area and other areas.

Study area	As	Cd	Cr	Cu	Pb	Zn	Reference
Jindo Island	0.040–0.200 (0.090)	0.004–0.070 (0.040)	0.020–0.160 (0.080)	0.120–0.500 (0.270)	0.010–0.070 (0.030)	0.120–0.570 (0.340)	This study
Jeju Island	0.020–0.050 (0.030)	0.001–0.050 (0.020)	0.010–0.120 (0.040)	0–0.170 (0.100)	0.009–0.030 (0.020)	0.090–0.450 (0.260)	This study
Gyeonggi Bay, Korea	NA	0.037–0.073 (0.048)	NA	0.460–1.020 (0.610)	0.010–0.035 (0.020)	0.140–1.190 (0.360)	Park et al. 2020b
Masan Bay, Korea	NA	0.007–0.027 (1.420)	NA	0.420–1.010 (0.660)	0.003–0.053 (0.012)	0.250–3.700 (1.420)	Park et al. 2020a
Yellow Sea, Korea	NA	0.015–0.041 (0.031)	NA	0.300–0.750 (0.500)	0.005–0.041 (0.014)	0.060–0.220 (0.15)	*United Nations Development Programme Global Environment Facility* 2007
Korean Marine Seawater Quality Standard	9.400	19.000	200.000	3.000	7.600	NA	Ministry of Oceans and Fisheries 2018

The varying correlations between heavy metals in seawater and environmental variables can be attributed to the distinct behaviors, sources, and sinks of different heavy metals (Zhang et al. 2018). On Jindo Island, Cr, and Pb showed positive correlations with suspended solids, while As was positively correlated with COD and IL (Figure 7a). Suspended solids in seawater act as both sinks and sources for heavy metals (Wang et al. 2011; Zhang et al. 2018). Conversely, off Jeju Island, As was positively correlated with COD and salinity, while Cr, Cd, and Zn were positively correlated with IL and salinity (Figure 7b). Suspended solids are a primary source of Cr and Cd in the seawater off Jindo Island. However, the mechanisms of migration and transformation of heavy metals in seawater and their interaction with organic matter in sediments vary under different environmental conditions (Geng et al. 2024). Seawater in Jindo Island primarily derives from organic matter in the sediments; similarly, off Jeju Island, As, Cr, Cd, and Zn originate from organic matter in sediments and are influenced by terrestrial freshwater input.

### 4.2 Distribution of Macrobenthos in the Subtidal Zones Off Jindo and Jeju Island

In our study, Annelida was the most prevalent taxonomic group in the subtidal zones off both islands. While Annelida also dominated the western seas of South Korea (Park et al. 2014), another study identified Cnidaria as the primary taxonomic group in the subtidal zone off southern Jeju Island (Ko et al. 2008).

In the subtidal zones off Jindo Island, *Heteromastus filiformis* exhibited the highest dominance, consistent with its common dominance in the western seas of South Korea (Park et al. 2014). In contrast, the subtidal zones of Jeju Island were dominated by Gammaridea, a suborder of Amphipoda. Amphipods are often considered indicator species due to their lower tolerance to pollution compared to annelids (Dauvin et al. 2016). This ecological distinction was evident in RDA (Figure 6), which showed a negative correlation between Gammaridea and heavy metal and organic matter concentrations, whereas *Heteromastus filiformis* was positively correlated with these environmental factors. The differences in heavy metal and organic matter levels between Jindo Island and Jeju Island likely contributed to the variation in dominant species observed in the two regions.

Overall, the subtidal zone off Jeju Island exhibited higher average species numbers and abundance compared to Jindo Island, with correspondingly greater values in the species richness index and the Shannon‐Wiener diversity index (Figure 6). This suggests that the macrobenthic communities in Jindo Island's subtidal zone may be under greater environmental stress, likely due to elevated heavy metal concentrations and increased organic matter content in the sediments, unlike the conditions found in Jeju Island.

### 4.3 Influence of Environmental Variables on Macrobenthic Communities

Our study revealed significant differences in species number, species abundance, species richness index, and Pielou's evenness index between the two islands (Figure 6). Moreover, cluster analysis, non‐metric MDS analysis, PERMANOVA, and PERMDISP showed variations in the structure of macrobenthic communities between the islands across 2012, 2010, and 2011 (Figure 4). The BIO‐ENV, dbRDA, and DistLM analyses identified the primary environmental variables affecting the macrobenthic community distribution as Cd and As between the islands, and Pb and Cr in the year 2012 compared to other years.

The average concentrations of As and Cd in the seawater off Jindo Island are three and two times higher, respectively, than those off Jeju Island. Spearman rank correlation analysis suggested a relationship between the concentrations of As and Cd off both islands' seawater and the organic matter in the sediments (Figure 7). As discussed in Section 4.1, the variation in As and Cd concentrations may be linked to riverine inputs, aquaculture activities, and seawater flow rates.

In 2012, the survey was conducted in September, while in 2010 and 2011 it took place in August. Normally, the flow of Changjiang diluted water into the southern seas of South Korea trails its initial discharge by 1–2 months (Moon et al. 2009). Consequently, the substantial influx of diluted water from the August monsoon season tends to impact the southern seas of South Korea starting in September (Kim et al. 2009). In the subtidal zones off Jindo Island, suspended solids displayed a negative correlation with salinity and a positive correlation with Cr and Pb. In contrast, in the subtidal zones off Jeju Island, Cr and IL were positively correlated with salinity, whereas Pb was negatively correlated with IL (Figure 7). These observations suggest that the diluted water from the Changjiang has influenced monthly variations in Cr and Pb concentrations, thereby affecting the structure of benthic communities.

Heavy metals typically have a negative impact on macrobenthic communities (Ryu et al. 2011). The toxicity varies with the type of metal and its various forms, which can influence its effects on macrobenthos (Dauvin 2008). For instance, in the subtidal zones off Jindo and Jeju island, Cd has been shown to negatively affect species diversity. However, an intriguing observation in the subtidal zones off Jindo Island is the positive correlation of Pielou's evenness index, the Shannon‐Wiener diversity index, and the Simpson index with certain heavy metals. We hypothesize that this could be attributed to varying tolerance levels among different macrobenthic species to heavy metals (Hu et al. 2019). In addition, the Intermediate Disturbance Hypothesis suggests that biodiversity often peaks under intermediate levels of disturbance (Valdivia et al. 2005).

### 4.4 Conservation Recommendations for the Coastal Areas Off South Korean Islands

With South Korea's economic growth, human activities have significantly impacted its coastal marine ecosystems. While the government has recognized the importance of marine conservation and implemented various protective measures since the 1990s, these ecosystems continue to face challenges. A notable shortfall is the lack of specific legislation to protect island marine environments. To comprehensively understand the long‐term impacts of human activities on these ecosystems, establishing sustained monitoring programs for macrobenthic communities in the subtidal zones of these islands is crucial.

In our study, although the concentrations of Cr and Cd in seawater were below Korea's Marine Seawater Quality Standards, they still adversely affected macrobenthos. This indicates that even low levels of heavy metals can impact marine organisms. Consequently, we recommend that the Korean government adopt more nuanced standards, such as the Threshold Effect Level or the Effect Range Low, to more accurately assess the ecological risks posed by heavy metals in seawater and enhance the protection of marine biodiversity.

## 5 Conclusions

In our study, 98 taxa were identified in the southern subtidal zone off Jindo island, while 104 taxa were identified in the northern subtidal zone off Jeju island. Differences exist between the two islands, and variations in macrobenthic communities are observed across different years. Our study indicates that the differences in macrobenthic communities between the islands are likely due to varying impacts of anthropogenic activities, whereas differences across years may be influenced by Changjiang diluted water.

Although the concentrations of heavy metals in the bottom waters off Jindo and Jeju island were below the South Korea Marine Seawater Quality Standard, Cd negatively impacted the macrobenthic communities of the two islands. We recommend that the Korean government establish a quality standard that addresses the effects of heavy metals on marine organisms in seawater. Our findings offer a valuable reference for protecting and managing marine ecosystems on South Korea's islands.

## Author Contributions

**Jian Liang:** conceptualization (equal), data curation (equal), formal analysis (equal), funding acquisition (equal), investigation (equal), methodology (equal), project administration (equal), resources (equal), software (equal), supervision (equal), validation (equal), visualization (equal), writing – original draft (equal), writing – review and editing (equal). **Chae‐Woo Ma:** resources (equal), software (equal), supervision (equal), validation (equal), visualization (equal), writing – review and editing (equal). **Kwang‐Bae Kim:** investigation (equal), resources (equal), software (equal), writing – original draft (equal), writing – review and editing (equal).

## Conflicts of Interest

The authors declare no conflicts of interest.

## Supporting information


Data S1.

## Data Availability

The raw data of this study can be found at https://doi.org/10.5061/dryad.3j9kd51vd.
