# Supplementary material for: Differences in Subtidal Macrobenthic Community Structures and Influencing Factors Between Jindo and Jeju Islands in South Korea
Source: Ecol Evol. 2025 Feb 24;15(2):e70990. doi: 10.1002/ece3.70990 (PMC11850444; doi:10.1002/ece3.70990)
Supplement: Supplementary file 1 — Data S1. [file ECE3-15-e70990-s001.docx]

*Supplementary Material*

Differences in subtidal macrobenthic community structures and influencing factors between two islands in South Korea

Jian Liang^1^, Chae-Woo Ma^1,^ * and Kwang-Bae Kim^2^

^1^ Department of Biology, College of Natural Sciences, Soonchunhyang University, Asan 31538, Republic of Korea

^2^Research Group of Tidal Flats, Gyeonggi-do Maritime and Fisheries Resources Research Institute, Ansan 15651, Republic of Korea

^*^ Correspondence: [cwooma@sch.ac.kr](mailto:cwooma@sch.ac.kr)

**Contents**

[Table S1 Coordinates of sampling sites. 2](#_Toc188197677)

[Table S2. The formulae for calculating the dominance and ecological indices. 3](#_Toc188197678)

[Table S3 Environment characteristics in the subtidal zones off Jindo island. 4](#_Toc188197679)

[Table S4 Environment characteristics in the subtidal zones off Jeju Island. 5](#_Toc188197680)

[Table S5. Eigenvectors of environmental factors with PC1 and PC2. 6](#_Toc188197681)

[Table S6. Eigenvectors of environmental factors to the dbRDA axes 1 and 2. 7](#_Toc188197682)

[Table S7 Results from BIO-ENV analyses for the subtidal zones off Jindo and Jeju island. 8](#_Toc188197683)

[Figure S1. Values of Species richness index (d) at each sampling site. Note: S1-S7, Sampling sites off Jindo island; S8-S14, Sampling sites off Jeju island. 9](#_Toc188197684)

[Figure S2. Values of Pielou’s evenness index(J') at each sampling site. Note: S1-S7, Sampling sites off Jindo island; S8-S14, Sampling sites off Jeju island. 10](#_Toc188197685)

[Figure S3. Values of Shannon-Wiener diversity index (H’) at each sampling site. Note: S1-S7, Sampling sites off Jindo island; S8-S14, Sampling sites off Jeju island. 11](#_Toc188197686)

[Figure S4. Values of Simpson index (1-Lambda’) at each sampling site. Note: S1-S7, Sampling sites off Jindo island; S8-S14, Sampling sites off Jeju island. 12](#_Toc188197687)

[Figure S5. Study area of Gyeonggi Bay, Massan Bay, Jindo Island, and Jeju Island in South Korea. 13](#_Toc188197688)

# Table S1 Coordinates of sampling sites.

| **Sampling site** | **Latitude** | **Longitude** |
| --- | --- | --- |
| S1 | 34˚ 21′ 29.7″ N | 126˚ 09′ 09.0″ E |
| S2 | 34˚ 19′ 29.4″ N | 126˚ 07′ 40.0″ E |
| S3 | 34˚ 20′ 28.6″ N | 126˚ 12′ 00.9″ E |
| S4 | 34˚ 20′ 22.0″ N | 126˚ 15′ 29.3″ E |
| S5 | 34˚ 19′ 31.4″ N | 126˚ 10′ 50.4″ E |
| S6 | 34˚ 16′ 47.6″ N | 126˚ 06′ 58.4″ E |
| S7 | 34˚ 17′ 08.7″ N | 126˚ 12′ 24.0″ E |
| S8 | 33˚ 34′ 24.1″ N | 126˚ 22′ 25.4″ E |
| S9 | 33˚ 30′ 32.9″ N | 126˚ 18′ 50.8″ E |
| S10 | 33˚ 31′ 52.3″ N | 126˚ 23′ 51.1″ E |
| S11 | 33˚ 33′ 45.9″ N | 126˚ 29′ 10.3″ E |
| S12 | 33˚ 29′ 34.0″ N | 126˚ 22′ 00.3″ E |
| S13 | 33˚ 29′ 59.0″ N | 126˚ 24′ 51.1″ E |
| S14 | 33˚ 31′ 09.5″ N | 126˚ 27′ 42.4″ E |

# Table S2. The formulae for calculating the dominance and ecological indices.

| **Indices** | **Algorithm** | **Note** |
| --- | --- | --- |
| Dominance index (Y) | = (ni/N) × fi, | ‘N’ denoted the total number of individuals across all species, ‘ni’ was the number of individuals of the ith species, and ‘fi’was the occurrence frequency of the ith species at study area |
| Species richness index (d) | $=(S-1)/\log(N)$ | S: The total number of species; N: The number of individual organisms. |
| Pielou’s evenness index(J') | $=H^{'}/\log(S)$ | H': Shannon-Wiener diversity index; S: The total number of species. |
| Simpson index (1-Lambda’) | $=1-\mathrm{SUM}\left( \mathrm{Ni}^{*}(\mathrm{Ni}-1)/\left( N^{*}(N-1) \right) \right.$ | Ni: Number of individuals of the ith species; N: The number of individual organisms. |
| Shannon-Wiener diversity index (H’) | $=-\sum\left[ \left( \frac{n_{i}}{N} \right)\log_{2}\left( \frac{n_{i}}{N} \right) \right]$ | Ni: Number of individuals belonging to the ith species; N: Total number of individuals. |

# Table S3 Environment characteristics in the subtidal zones off Jindo island.

| **Environment Factors** | **Range (min-max)** | **Mean ± CV** |
| --- | --- | --- |
| AVS, mg/g | 0-0.007 | 0.003±0.77 |
| COD, mg/kg | 1558.48-6630.07 | 4040.19±0.43 |
| DO, mg/L | 6.94-9.02 | 7.60±0.10 |
| IL, % | 2.54-6.27 | 4.29±0.27 |
| Mean grain size, ∮ | 2.00-7.60 | 4.55±0.34 |
| pH | 7.93-8.13 | 8.08±0.01 |
| Salinity, PSU | 31.19-32.64 | 31.98±0.01 |
| Suspended Solids, mg/L | 20.40-47.60 | 30.20±0.28 |
| Total Nitrogen, mg/L | 0.20-0.54 | 0.39±0.25 |
| Total Phosphorus, mg/L | 0.01-0.04 | 0.03±0.22 |
| Water temperature, ℃ | 18.90-23.20 | 21.08±0.05 |
| As, ㎍/L | 0.04-0.20 | 0.094±0.22 |
| Cd, ㎍/L | 0.004-0.07 | 0.036±0.55 |
| Cr, ㎍/L | 0.02-0.16 | 0.078±0.55 |
| Cu, ㎍/L | 0.12-0.50 | 0.266±0.41 |
| Pb, ㎍/L | 0.01-0.07 | 0.035±0.47 |
| Zn, ㎍/L | 0.12-0.57 | 0.343±0.38 |

Note: AVS, acid-volatile sulfide; COD, chemical oxygen demand; DO, dissolved oxygen; IL, ignition loss; CV, Coefficient of variation.

# Table S4 Environment characteristics in the subtidal zones off Jeju Island.

| **Environment Factors** | **Range (min-max)** | **Mean ± CV** |
| --- | --- | --- |
| AVS, mg/g | 0-0.004 | 0.002±0.52 |
| COD, mg/Kg | 836.23-7185.04 | 3625.55±0.59 |
| DO, mg/L | 6.87-8.05 | 7.41±0.06 |
| IL, % | 1.20-5.10 | 3.37±0.41 |
| Mean grain size, ∮ | 0.90-4.80 | 2.84±0.46 |
| pH | 7.86-8.23 | 8.09±0.01 |
| Salinity, PSU | 28.71-33.56 | 31.67±0.07 |
| Suspended Solids, mg/L | 16.20-46.00 | 27.55±0.30 |
| Total Nitrogen, mg/L | 0.20-0.80 | 0.43±0.30 |
| Total Phosphorus, mg/L | 0.01-0.04 | 0.03±0.29 |
| Water temperature, ℃ | 17.20-23.50 | 20.61±0.10 |
| As, ㎍/L | 0.02-0.05 | 0.026±0.42 |
| Cd, ㎍/L | 0.001-0.05 | 0.017±1.16 |
| Cr, ㎍/L | 0.01-0.12 | 0.038±0.75 |
| Cu, ㎍/L | 0-0.17 | 0.101±0.45 |
| Pb, ㎍/L | 0.009-0.03 | 0.018±0.27 |
| Zn, ㎍/L | 0.09-0.45 | 0.255±0.35 |

Note: AVS, acid-volatile sulfide; COD, chemical oxygen demand; DO, dissolved oxygen; IL, ignition loss; CV, Coefficient of variation.

# Table S5. Eigenvectors of environmental factors with PC1 and PC2.

| **Environment Factors** | **PC1** | **PC2** |
| --- | --- | --- |
| AVS, mg/g | -0.094 | 0.265 |
| COD, mg/g | 0.378 | -0.063 |
| DO, mg/L | -0.306 | 0.246 |
| IL, % | 0.385 | 0.069 |
| Mean grain size, ∮ | -0.095 | 0.250 |
| pH | 0.036 | 0.052 |
| Salinity, PSU | 0.364 | -0.035 |
| Suspended Solids, mg/L | -0.277 | 0.269 |
| Total Nitrogen, mg/L | 0.044 | -0.261 |
| Total Phosphorus, mg/L | 0.342 | -0.014 |
| Water temperature, ℃ | 0.347 | -0.048 |
| As, ㎍/L | 0.204 | 0.252 |
| Cd, ㎍/L | 0.228 | 0.239 |
| Cr, ㎍/L | 0.123 | 0.396 |
| Cu, ㎍/L | 0.116 | 0.365 |
| Pb, ㎍/L | -0.006 | 0.396 |
| Zn, ㎍/L | 0.157 | 0.289 |

Note: AVS, acid-volatile sulfide; COD, chemical oxygen demand; DO, dissolved oxygen; IL, ignition loss.

# Table S6. Eigenvectors of environmental factors to the dbRDA axes 1 and 2.

| **Environment Factors** | **dbRDA1** | **dbRDA2** |
| --- | --- | --- |
| AVS, mg/g | -2.98 | -5.48 |
| COD, mg/g | 12.91 | 15.12 |
| DO, mg/L | 19.98 | 10.73 |
| IL, % | 1.71 | -4.30 |
| Mean grain size, ∮ | 1.39 | 2.39 |
| pH | 1.91 | -1.41 |
| Salinity, PSU | -3.75 | -4.84 |
| Suspended Solids, mg/L | -0.35 | 3.04 |
| Total Nitrogen, mg/L | 0.22 | 0.20 |
| Total Phosphorus, mg/L | 1.72 | 0.41 |
| Water temperature, ℃ | 0.17 | 1.48 |
| As, ㎍/L | 5.76 | -0.78 |
| Cd, ㎍/L | 12.45 | -5.02 |
| Cr, ㎍/L | -5.14 | -6.25 |
| Cu, ㎍/L | 2.35 | 5.34 |
| Pb, ㎍/L | -5.00 | 6.63 |
| Zn, ㎍/L | 4.32 | 1.50 |

Note: AVS, acid-volatile sulfide; COD, chemical oxygen demand; DO, dissolved oxygen; IL, ignition loss.

# Table S7 Results from BIO-ENV analyses for the subtidal zones off Jindo and Jeju island.

| **Study area** |  | **Number of environmental variables** | **Correlation coefficient** | **Environmental variables** |
| --- | --- | --- | --- | --- |
| Jindo Island |  | 4 | 0.339 | Water temperature, pH, Pb, Mean grain size |
|  |  | 5 | 0.339 | Water temperature, pH, As, Pb, Mean grain size |
|  |  | 6 | 0.327 | Water temperature, pH, As, Pb, Zn, Mean grain size |
| Jeju Island |  | 2 | 0.643 | Cr, Cd |
|  |  | 1 | 0.638 | Cr |
|  |  | 3 | 0.627 | Cr, Cd, Cu |
| Total |  | 3 | 0.409 | As, Cd, Pb |
|  |  | 4 | 0.392 | As, Cd, Cu, Pb |
|  |  | 2 | 0.391 | As, Cd |


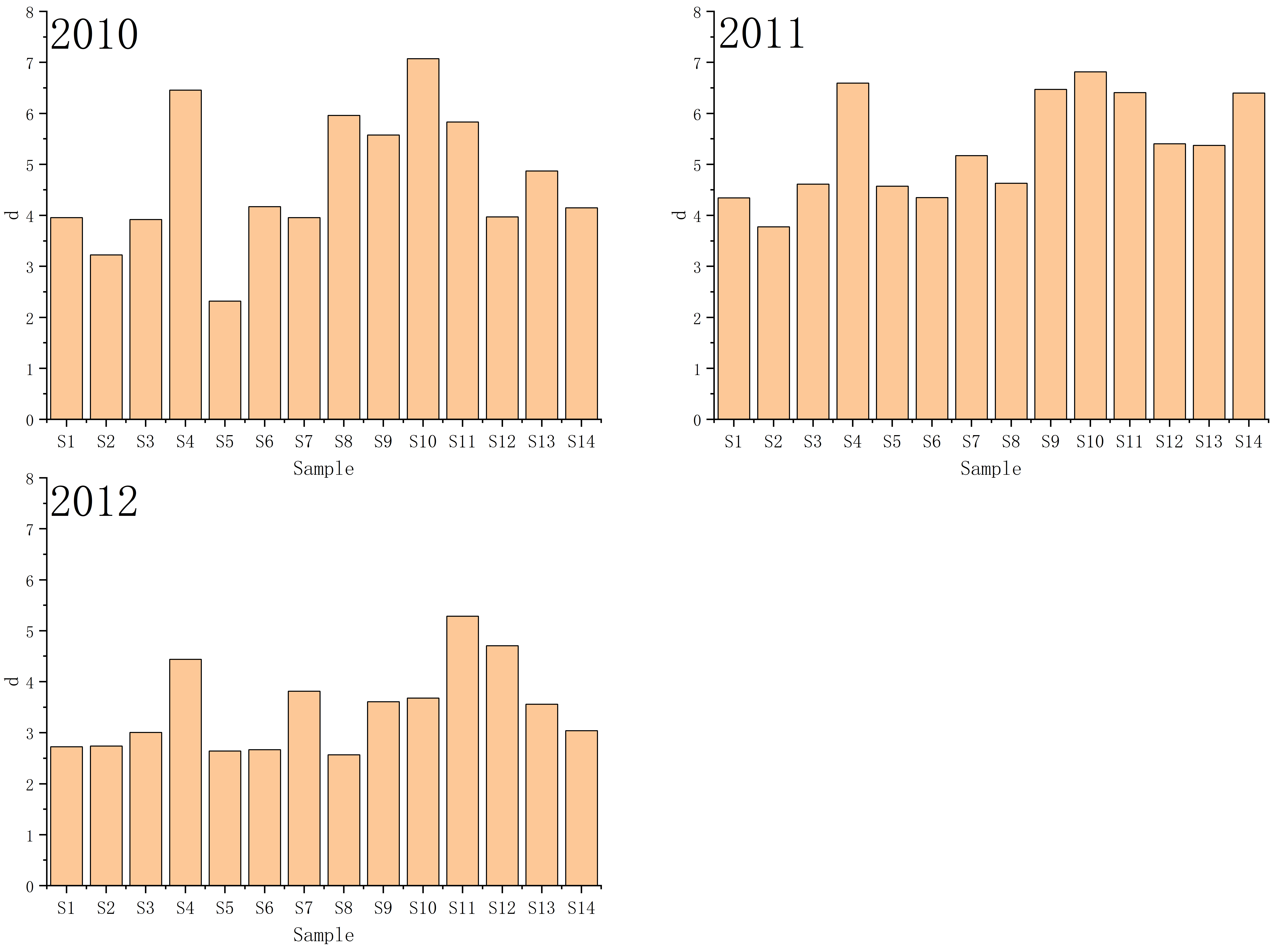


# Figure S1. Values of Species richness index (d) at each sampling site. Note: S1-S7, Sampling sites off Jindo island; S8-S14, Sampling sites off Jeju island.


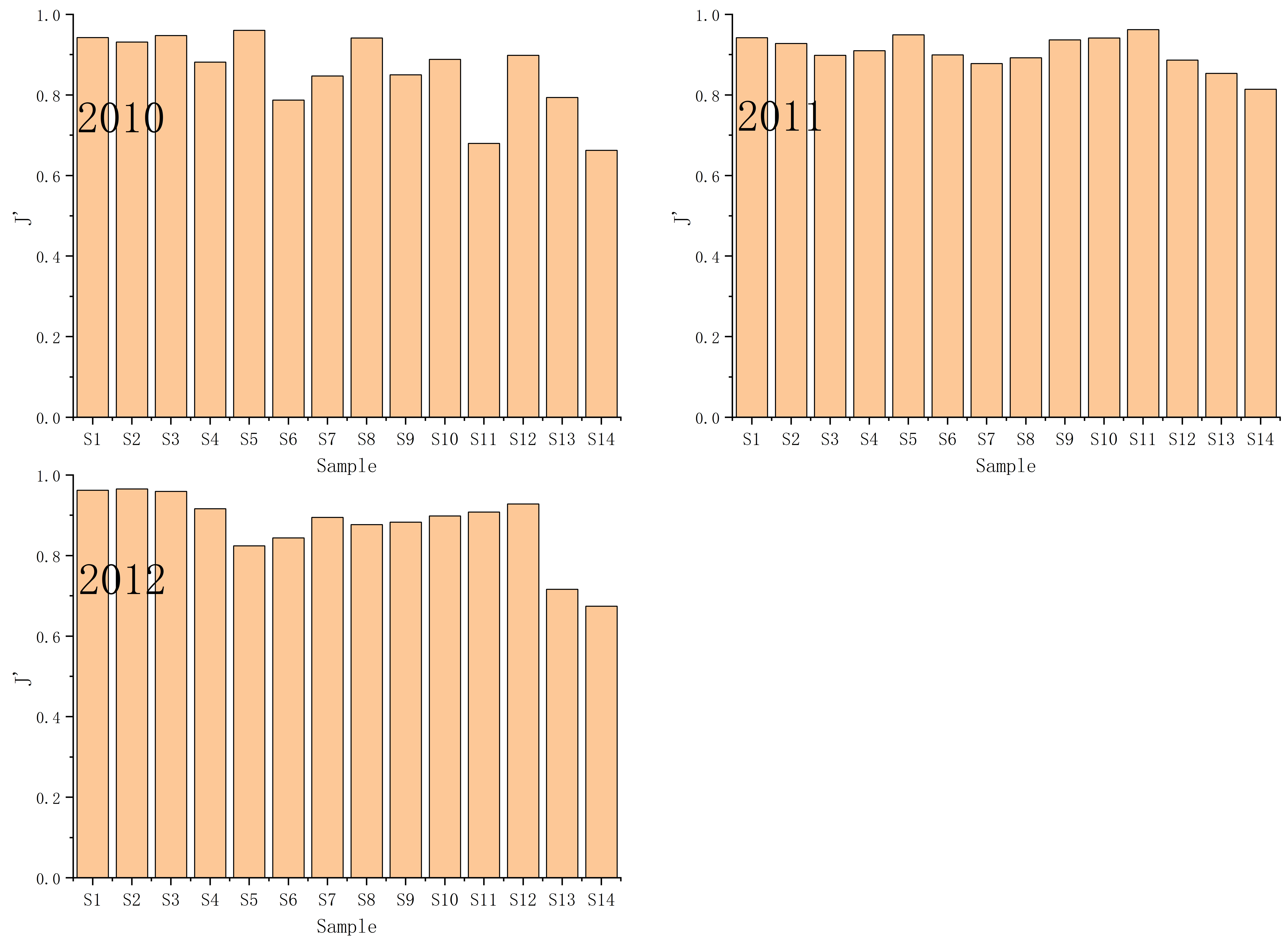


# Figure S2. Values of Pielou’s evenness index(J') at each sampling site. Note: S1-S7, Sampling sites off Jindo island; S8-S14, Sampling sites off Jeju island.


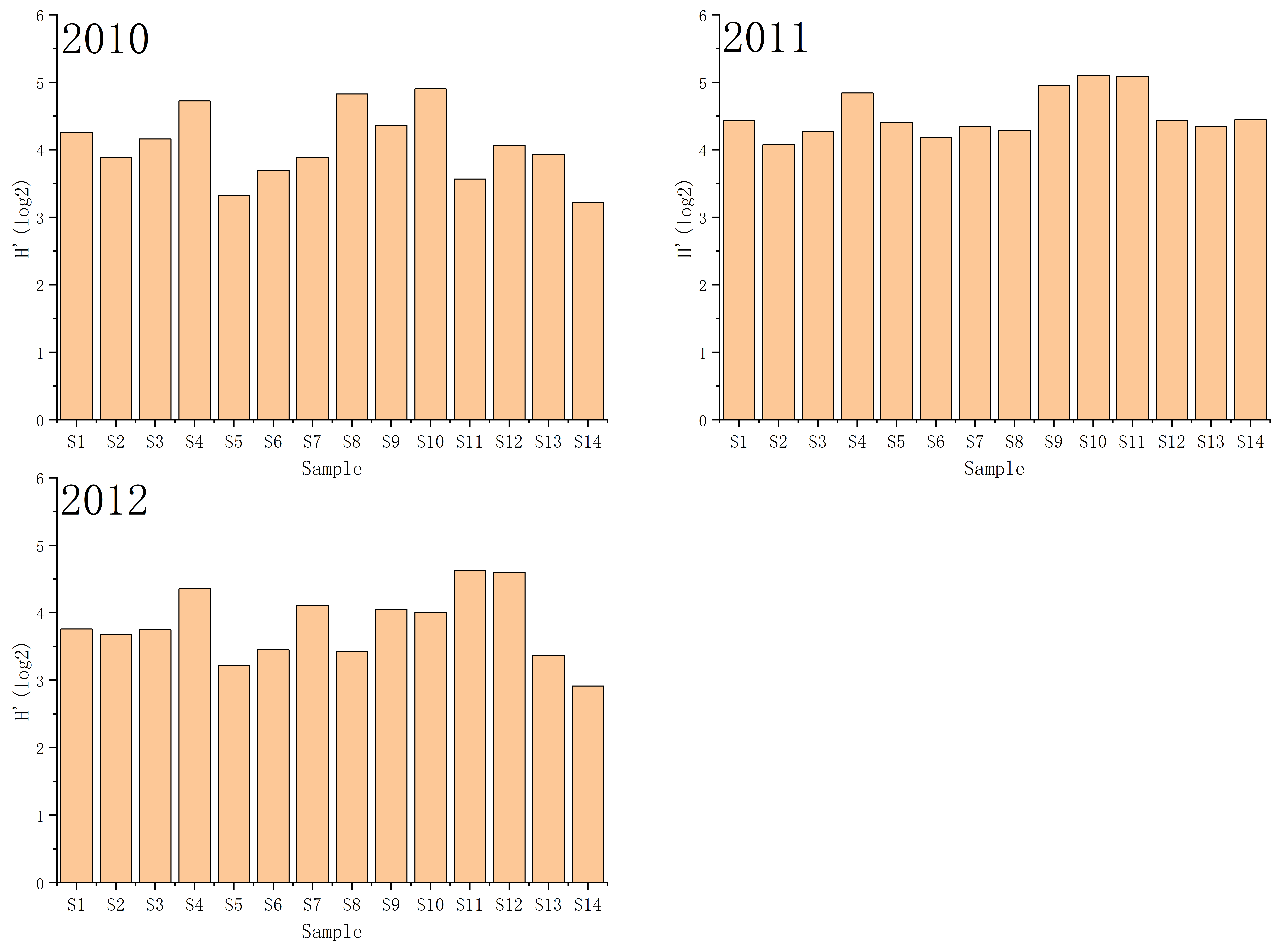


# Figure S3. Values of Shannon-Wiener diversity index (H’) at each sampling site. Note: S1-S7, Sampling sites off Jindo island; S8-S14, Sampling sites off Jeju island.


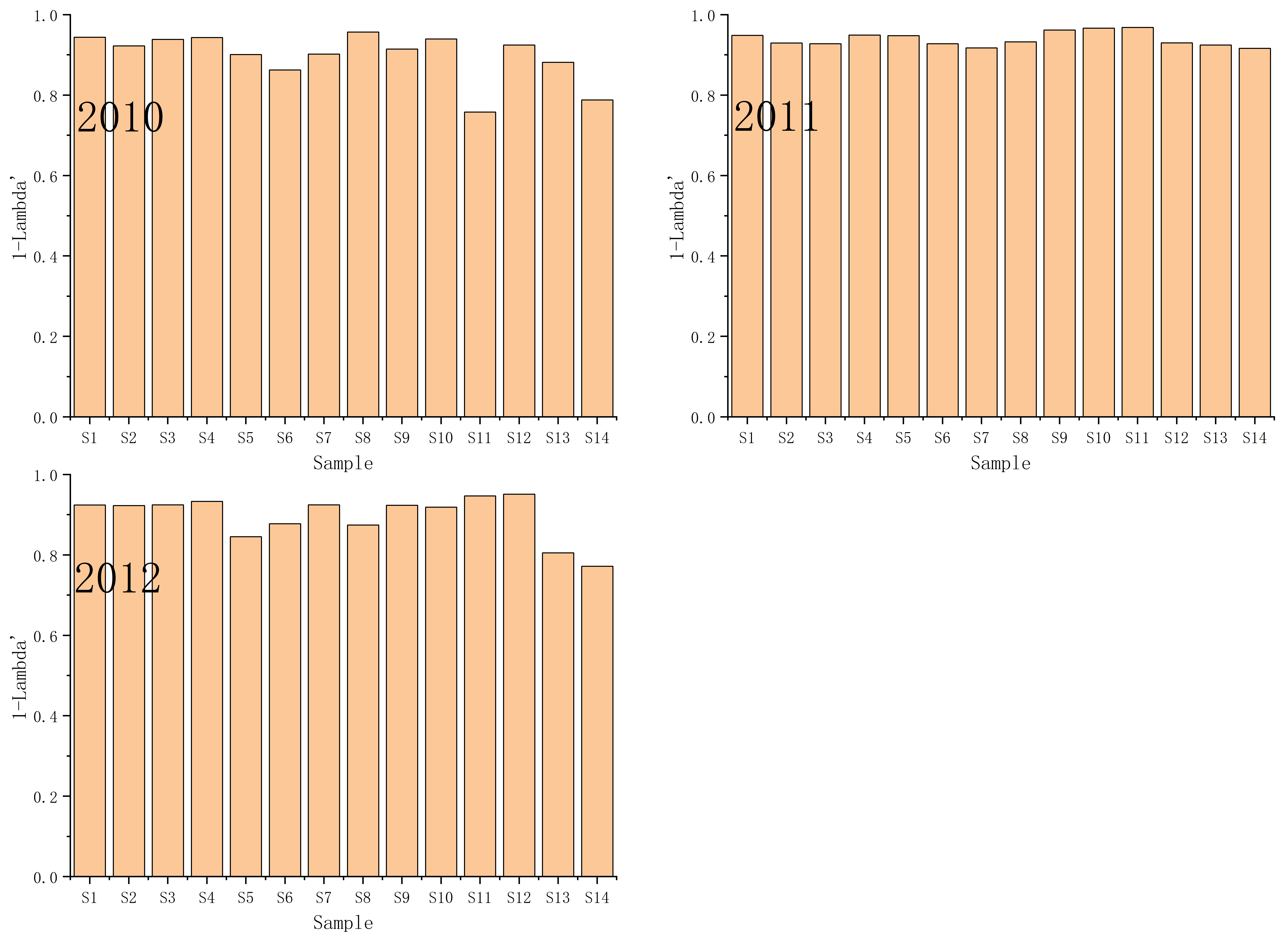


# Figure S4. Values of Simpson index (1-Lambda’) at each sampling site. Note: S1-S7, Sampling sites off Jindo island; S8-S14, Sampling sites off Jeju island.


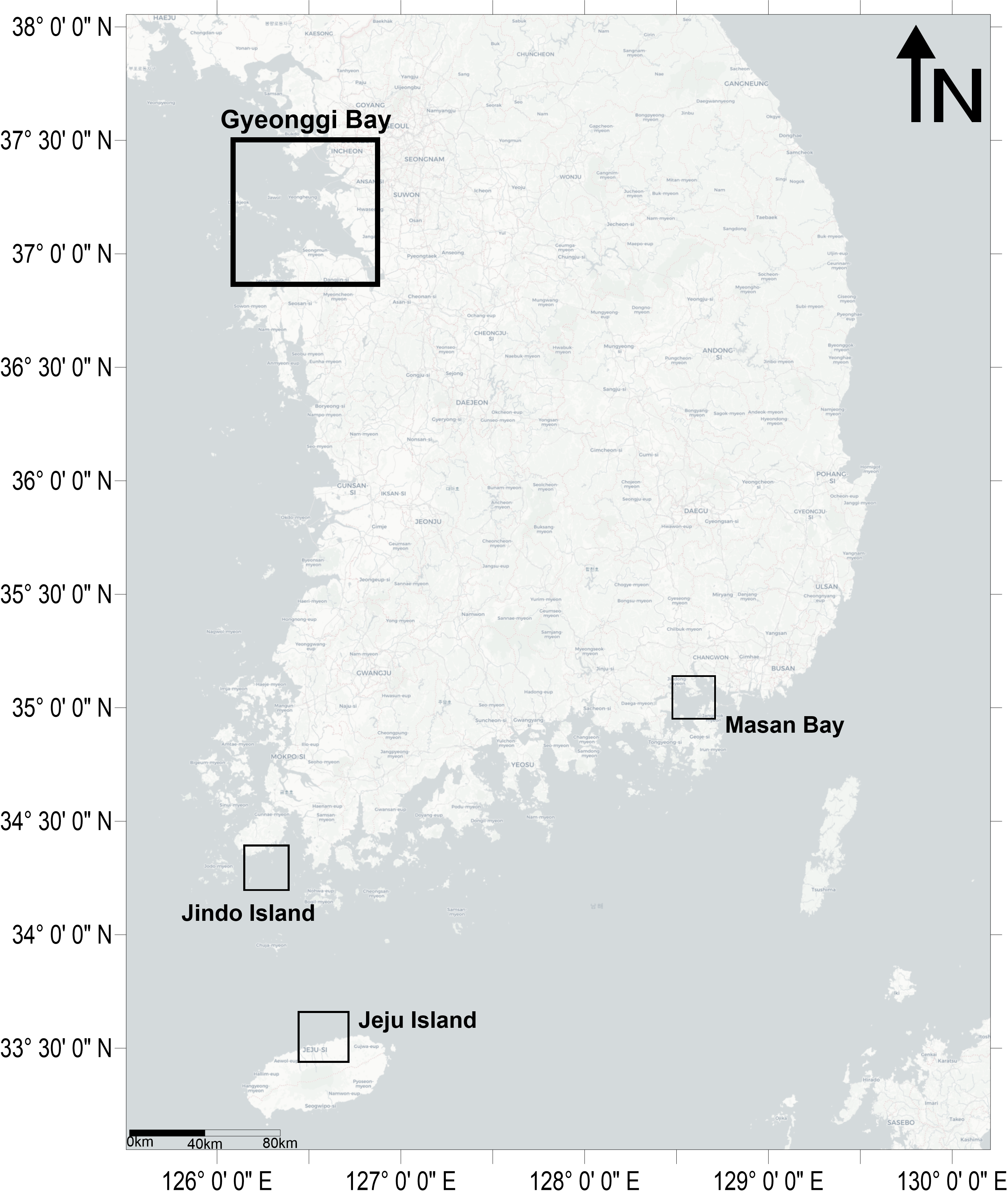


# Figure S5. Study area of Gyeonggi Bay, Massan Bay, Jindo Island, and Jeju Island in South Korea.
